# An approach to psychosocial health among middle-aged and older people by remote sharing of photos and videos from family members not living together: A feasibility study

**DOI:** 10.3389/fpubh.2022.962977

**Published:** 2022-11-10

**Authors:** Taiji Noguchi, Michi Sato, Tami Saito

**Affiliations:** ^1^Department of Social Science, Center for Gerontology and Social Science, National Center for Geriatrics and Gerontology, Research Institute, Obu, Japan; ^2^Japan Society for the Promotion of Science, Chiyoda, Japan; ^3^Chikaku Inc., Shibuya, Japan

**Keywords:** digital intervention, family satisfaction, feasibility, psychosocial health, remote communication, social relationships

## Abstract

**Background:**

As an approach to the psychosocial health of people in later adulthood, information and communication technology (ICT) is attracting attention. However, because there is still a disparity issue in ICT use, particularly for older people, considering age-friendly digital interventions is important. We examined the feasibility of an intervention by an age-friendly digital service, remote sharing of photos/videos from families not living together, for psychosocial health in middle-aged and older people.

**Methods:**

This single-arm study recruited Japanese adults aged ≥ 50 years from web-pages of the social service, Mago-Channel (Chikaku Inc., Japan). Participants used this service for 3 months to receive photos/videos from the smartphones of their families not living together on a device set up on their home TVs and watched them there. Families not living together were encouraged to send photos/videos at least once a week, but no other restrictions on their lives, including their interactions, were imposed. After 3 months, the level of user satisfaction and changes in psychosocial health were assessed.

**Results:**

Finally, 115 participants were included, and 106 completed the intervention; the dropout rate from the intervention was low (7.8%), and satisfaction with the program was high, indicating high feasibility. While depressive symptoms and loneliness did not change markedly, satisfaction with the relationship of families living together increased significantly, and social interactions improved, including those with families not living together.

**Conclusions:**

High feasibility of this age-friendly digital intervention and its potential benefits on social relationships were shown, encouraging further trials with a confirmatory study design.

## Introduction

Psychosocial health of people in later adulthood is becoming more important with global aging. After middle and old age, people experience narrowing social relationships, such as separation from their children, widowhood, and decreased friendships (1). Particularly, in Japan, where population aging is rapidly proceeding, the rate of older adults reached 28.9% as of 2021 (2). Additionally, due to the increasing prevalence of nuclear families and never-married individuals (3), the number of people socially isolated in later adulthood is rapidly expanding (4). Because both social and biomedical knowledge suggests that social relationships are fundamentally important for mental health (5, 6), ensuring social interactions is crucial for people in later life.

In order to support social connectedness of people in later adulthood, information and communication technology (ICT) use is attracting attention. In particular, the recent novel coronavirus disease 2019 (COVID-19) pandemic (7) limited in-person social interactions among people, which triggered a decline in mental health (8, 9). This social restriction highlighted the value of remote connections between people, including online connections (10, 11). Indeed, remote communication *via* Internet use has the potential to improve the mental health of people in later life (12, 13) and to encourage participation in social activities (13). However, some people, particularly older people, may be left out of this trend. Even though the number of middle-aged and older people using the Internet and digital social media has increased (14, 15), it is still much lower than that of younger people in most countries (16, 17). People in later adulthood, including older people, may face barriers to digital engagement, raising the issue of the “digital divide” owing to physical and cognitive functioning, financial issues, the culture of communication, fear, or low motivation (17). Hence, considering age-friendly digital approaches to enhance remote social connections of people in later adulthood is required.

Recently, a social service, “Mago-Channel” (Chikaku Inc., Japan), has been launched that promotes ICT-based remote social relationships among middle-aged and older people (“Mago” means “grandchild” in Japanese) (18, 19). This service allows families not living together, including their children and grandchildren, to share their photos and videos with middle-aged and older people. They can then easily view the photos and videos received on their home TV. Here, the “family not living together” mainly includes their children and grandchildren, regardless of how far away they live; in Japan, owing to the spread of nuclear families, many middle-aged and older people live apart from their children and grandchildren. This age-friendly digital service may be a new approach to help people in later adulthood interact with others outside families living together and support their psychosocial health.

As with several efforts reported in the past (20–22), informal and simple forms of social interaction through sharing photos and videos may be beneficial as an age-friendly digital tool. Photos and videos are the main sources of interaction with family and friends. Viewing past photos and videos brings back memories. Watching children and grandchildren grow up through photos and videos fosters parents' and grandparents' generativity, which can also promote their wellbeing. Moreover, it has the potential to facilitate communication with families and friends.

This study aimed to investigate the feasibility of an intervention in psychosocial health in middle-aged and older people through remote sharing of photos and videos from families not living together, based on the use of the Mago-Channel. In particular, we examined the feasibility of the intervention in terms of dropout rate during the study period and satisfaction with the intervention program. Additionally, we evaluated changes in psychological health and social relationships with families living together and not together, as well as with friends, during the intervention period.

## Methods

### Study participants

This single-arm study recruited Japanese adults aged ≥ 50 years. The entry gate for study participation was set up on the web page of the Mago-Channel product. The exclusion criteria were those aged < 50 years, those who could not use the service owing to difficulties setting up the Mago-Channel, such as not having a TV at home, and those with difficulties receiving photos and videos owing to families not living together who did not have smartphones or who had difficulties sending them at least once a week.

**Figure 1:**
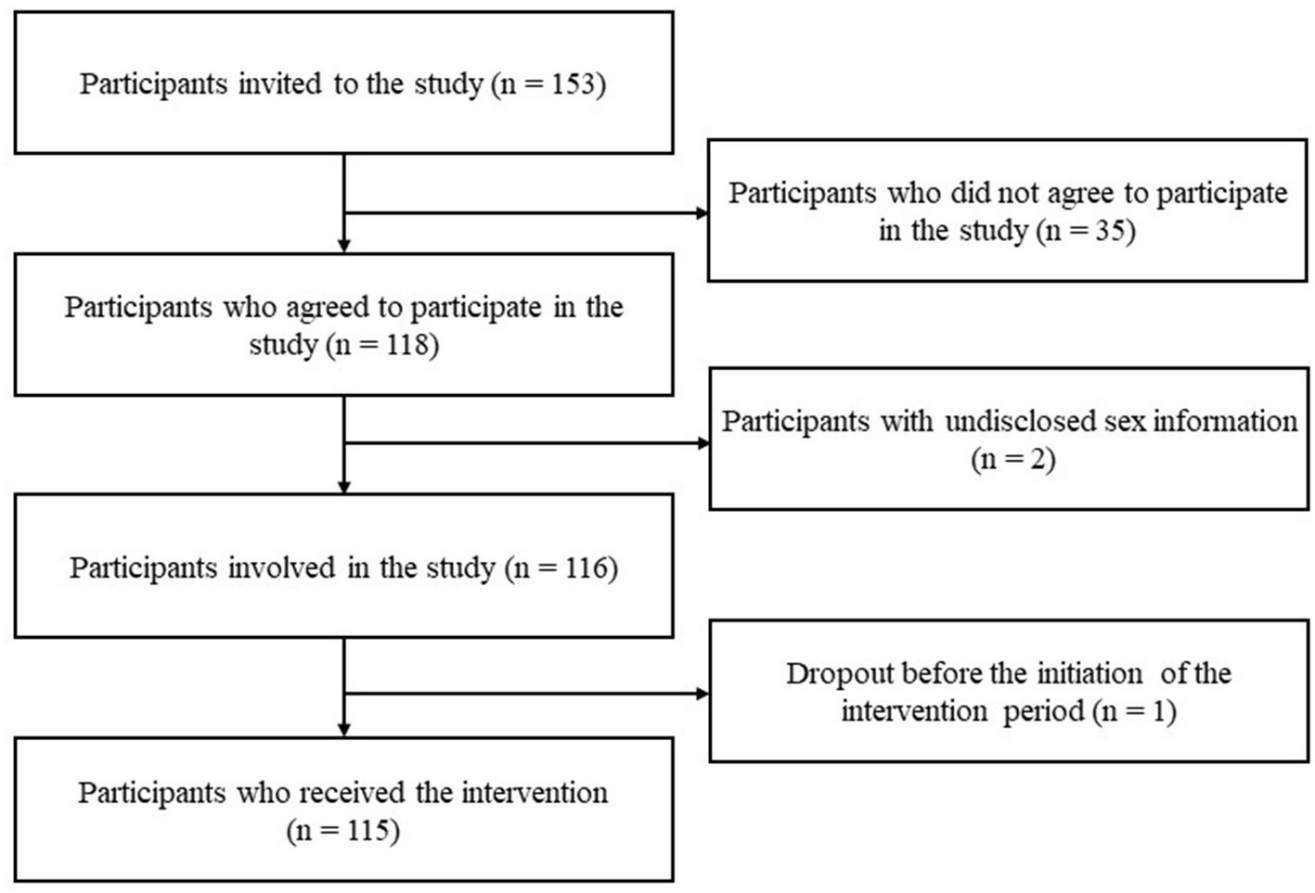
Sampling flow chart.

Figure 1 shows the sample selection flow. Between September 2020 and May 2021, 153 people applied to this study. Of these, 35 declined before giving consent to the study, two were excluded owing to undisclosed sex information, and one dropped out before the intervention. Therefore, 115 participants received the intervention program and questionnaire-based assessment. Additionally, people who sent photos and videos using the Mago-Channel to middle-aged and older participants, such as their family members not living with them (called “senders”), were also included as study participants and completed a mailed questionnaire at the end of the intervention period.

This study was reviewed and approved by the Research Ethics Committee of the National Center for Geriatrics and Gerontology (No. 1375 and 1419). Informed consent was obtained by mail, with a written explanation of the study and a returned reply of written consent from the participants. This study was conducted in conformance with the provisions of the Declaration of Helsinki. This study was registered with the University Hospital Medical Information Network (UMIN) clinical trial registry (No. UMIN000041213).

### Procedure

Participants were provided with the service of the Mago-Channel (Chikaku Inc., Tokyo, Japan) as an intervention. This ICT-based social service allows others, such as families not living together or friends, to send their photos and videos *via* their smartphones to the participants' homes after installing a special application, and allows participants to watch these photos and videos on their TV. To use the service, participants set up a small device (“receiver box”) in their home and connected it to their TV. As the communication lines are built into the device, the Internet or a wireless LAN is not needed for its use. After completing these procedures, using the TV remote control, participants could watch photos and videos from their families not living with them sent to the participants' receiver boxes on their TV.

Participants used this service for 3 months. The senders, that is, the families not living together, were encouraged to share photos and videos at least once a week. Participants and their families not living together had no other restrictions in their daily lives, such as talking on the phones or by email, or going to and from each other's homes.

Middle-aged and older participants answered a questionnaire at baseline and then during the first, second, and third months from the start of the intervention. The senders answered a questionnaire about satisfaction and usability at the end of the intervention. The data were collected and analyzed only by the investigators, not through Chikaku Inc.

### Measurement

#### Feasibility outcome

The following three items were assessed for the feasibility of the intervention program: dropout rate during the intervention period, frequency of service usage, and user satisfaction. Additionally, senders' evaluations of the service were assessed.

##### Dropout rate

The dropout rate during the intervention period was used as a primary endpoint. Dropouts were defined as service termination, study withdrawal, or no use of the service for over a month.

##### Frequency of service usage

The frequency of service usage daily was calculated from the recorded access data to the Mago-Channel.

##### User satisfaction

For middle-aged and older adult participants, satisfaction with use of the services was assessed with the following question at the end of the intervention: “Please rate Mago-Channel satisfaction out of 10.” Users' satisfaction was scored on a 0–10-point scale.

##### Sender's evaluations of the service assessment

For the senders, such as family members not living together, the following four items were assessed at the end of the intervention: users' satisfaction, users' enjoyment level, usability, and users' burden. Users' satisfaction was assessed with the question, “Please rate Mago-Channel satisfaction out of 10,” scored on a 0–10-point scale. Users' enjoyment level was assessed with the question, “Did you enjoy using the Mago-Channel?” (Possible answers: “strongly agree,” “agree,” “neither,” “disagree,” or “strongly disagree.”) Usability was assessed with the question, “Was the Mago-Channel easy to use?” (Possible answers: “very usable,” “usable,” “neither,” “unusable,” “very unusable.”). Users' burden was assessed with the question, “Did you have any burden to send photos or videos *via* the Mago-Channel?” (Possible answers: “almost none,” “not much,” “neither,” “a little,” or “quite”).

#### Psychosocial health

Psychosocial health was assessed at four time-points: the baseline, and the first, second, and third months, based on the questionnaire.

##### Depressive symptoms

Depressive symptoms were assessed using the Japanese version of the Center for Epidemiologic Studies Depression Scale (CES-D) (23). The CES-D is a validated assessment that contains 20 questions (0–3 points, respectively) and is rated from 0–60 points. Higher scores indicate depressive tendencies. Clinically, a score of 16 or above is used as a cut-off point (23). Cronbach's α was 0.83 in the baseline data, indicating good internal consistency.

##### Loneliness

Loneliness was assessed using the Japanese version of the UCLA Loneliness Scale (24). The UCLA Loneliness Scale is a validated scale containing 20 questions (1–4 points, respectively) and rated from 20–80 points. Higher scores indicate higher feelings of loneliness. Cronbach's α was 0.80 in the baseline data, indicating good internal consistency.

##### Satisfaction with the relationship of family members living together

Satisfaction with the relationship of family members living together was assessed by the question, “Rate your current satisfaction of relationships with your families living together on 0–10 points”.

##### Talking time with family members living together

The daily talking time with family members living together was assessed using the question, “How much time a day do you spend talking with family members who live with you?” (Possible answers: “rarely,” “ <30 min/day,” “30 to 60 min/day,” “60 to 120 min/day,” or “more than 120 min/day.”). To quantify the length of time change during the intervention period, we used the responses as a continuous variable by converting them into daily time (min/day) as follows: “rarely,” = 0; “ <30 min/day” = 15; “30 to 60 min/day” = 45; “60 to 120 min/day” = 90; “more than 120 min/day” = 120.

##### Frequency of talking with family members not living together

The frequency of talking with family members not living together was assessed using the question, “How often do you talk with your immediate family members who do not live together, including phone calls and emails?” (Possible answers: “none,” “a few times a year,” “once or twice a month,” “once a week,” “two or three times a week,” or “almost every day.”) We used the responses as a continuous variable by converting them into monthly frequency (times/month), based on a period of 4.3 weeks per month (25, 26), as follows: “none” = 0; “a few times a year” = 0.2; “once or twice a month” = 1.5; “once a week” = 4.3; “two or three times a week” = 10.8; “almost every day” = 21.5.

##### Frequency of talking with friends

The frequency of talking with friends was asked using the question, “How often do you talk with your friends, including phone calls and emails?” (Possible answers: “none,” “a few times a year,” “once or twice a month,” “once a week,” “two or three times a week,” or “almost every day.”) We used the responses as a continuous variable by converting them into monthly frequency (times/month), based on a period of 4.3 weeks per month (25, 26), as follows: “none” = 0; “a few times a year” = 0.2; “once or twice a month” = 1.5; “once a week” = 4.3; “two or three times a week” = 10.8; “almost every day” = 21.5.

#### Interactions with others based on service use

At the end of the intervention, participants were assessed for their interactions with others relating to the use of the Mago-Channel during the study period. Participants were asked whether they had talked with family members living together, family members not living together, and friends, about the photos and videos received on the Mago-Channel, respectively (possible answers: “very often,” “often,” “a little” or “rarely”).

#### Other variables

Participants' age, sex, living arrangement, mental illnesses, psychotropic drug use, visual impairment, and daily use of email or social networking services (SNS) were assessed at baseline.

### Statistical analysis

First, the dropout rate during the intervention period was calculated, and descriptive statistics for user satisfaction and the number of uses per day were calculated for intervention feasibility. Additionally, the senders' satisfaction, enjoyment level, usability, and burden were calculated for feasibility. Next, changes in psychosocial health indicators during the intervention were estimated using a linear mixed-effects model (time as a fixed effect; individual as a random effect), and regression coefficients (βs) and standard errors (SEs) were obtained for the psychosocial health indicators.

To explore psychosocial health changes of different analyzed groups, we performed *post hoc* analyses restricted to the following groups: those without mental illnesses and taking psychotropic drugs, without low vision, living alone, aged ≥ 75 years, male sex, rarely using email or SNS at baseline, and having fewer interactions with their families not living together at baseline; these often talked with their families living together, families not living together, and friends about the photos and videos received during the intervention, and revived photos and videos continuously for the intervention.

The significance level was set at p < 0.05. All analyses were conducted using R (Version 4.0.3 for Windows; R Foundation for Statistical Computing, Austria).

## Results

A total of 115 participants received the intervention. Table 1 shows the characteristics of participants at baseline. The participants' mean age (standard deviation: SD) was 74.3 (9.6) years, and 57.4% were female participants. Of the participants, 26 (22.6%) were living alone, 6 (5.3%) had mental illnesses, 9 (7.9%) were taking psychotropic drugs, and 16 (13.9%) had low visual function; 64 (55.7%) used email or SNS on a daily basis. Regarding psychosocial health, the mean scores (SD) were 13.2 (7.5) for the CES-D, 37.6 (10.1) for the UCLA Loneliness Scale, and 7.2 (2.0) for satisfaction with the relationship of families living together; 30.9% of the participants had a score above the cut-off point (16 or above) for the CES-D. Participants spent an average of 72.1 (SD: 44.5) minutes/day talking with family members living together, had an average of 9.2 (7.4) times/month talking with family members not living together, and had 7.6 (7.4) times/month talking with friends.

**Table 1:** Characteristics of participants at baseline.

		***n =* 115**
		*N* (%)
Sex	Men	49 (42.6)
	Women	66 (57.4)
Living arrangement	Living with others	89 (77.4)
	Living alone	26 (22.6)
Mental illness	No	107 (94.7)
	Yes	6 (5.3)
Psychotropic drug use	No	105 (92.1)
	Yes	9 (7.9)
Visual function	Normal	99 (86.1)
	Low	16 (13.9)
Daily use of email or social network service	No	51 (44.3)
	Yes	64 (55.7)
		Mean (SD)
Age (years)		74.3 (9.6)
CES-D score		13.2 (7.5)
UCLA Loneliness Scale score		37.6 (10.1)
Satisfaction score of the relationship with families living together^*^		7.2 (2.0)
Talking time with families living together* (min/day)		72.1 (44.5)
Frequency of talking to families not living together (times/month)		9.2 (7.4)
Frequency of talking to friends (times/month)		7.6 (7.4)

CES-D, The Center for Epidemiologic Studies Depression Scale; SD, standard deviation.

* Exclude those living alone (n = 26).

Missing data: mental illness, n = 2; psychotropic drug use, n = 1; CES-D score, n = 14; UCLA Loneliness Scale score, n = 19; satisfaction score for families living together, n = 7; taking time with families living together, n = 2; frequency of talking to families not living together, n = 1.

During the intervention period, nine dropped out of the study (dropout rate: 7.8%): one canceled the service of Mago-Channel, four withdrew from the study, and four had not used the service for over a month. The reasons for dropouts were not related to the intervention content, and were largely attributed to the burden of answering the questionnaire for assessment.

**Figure 2:**
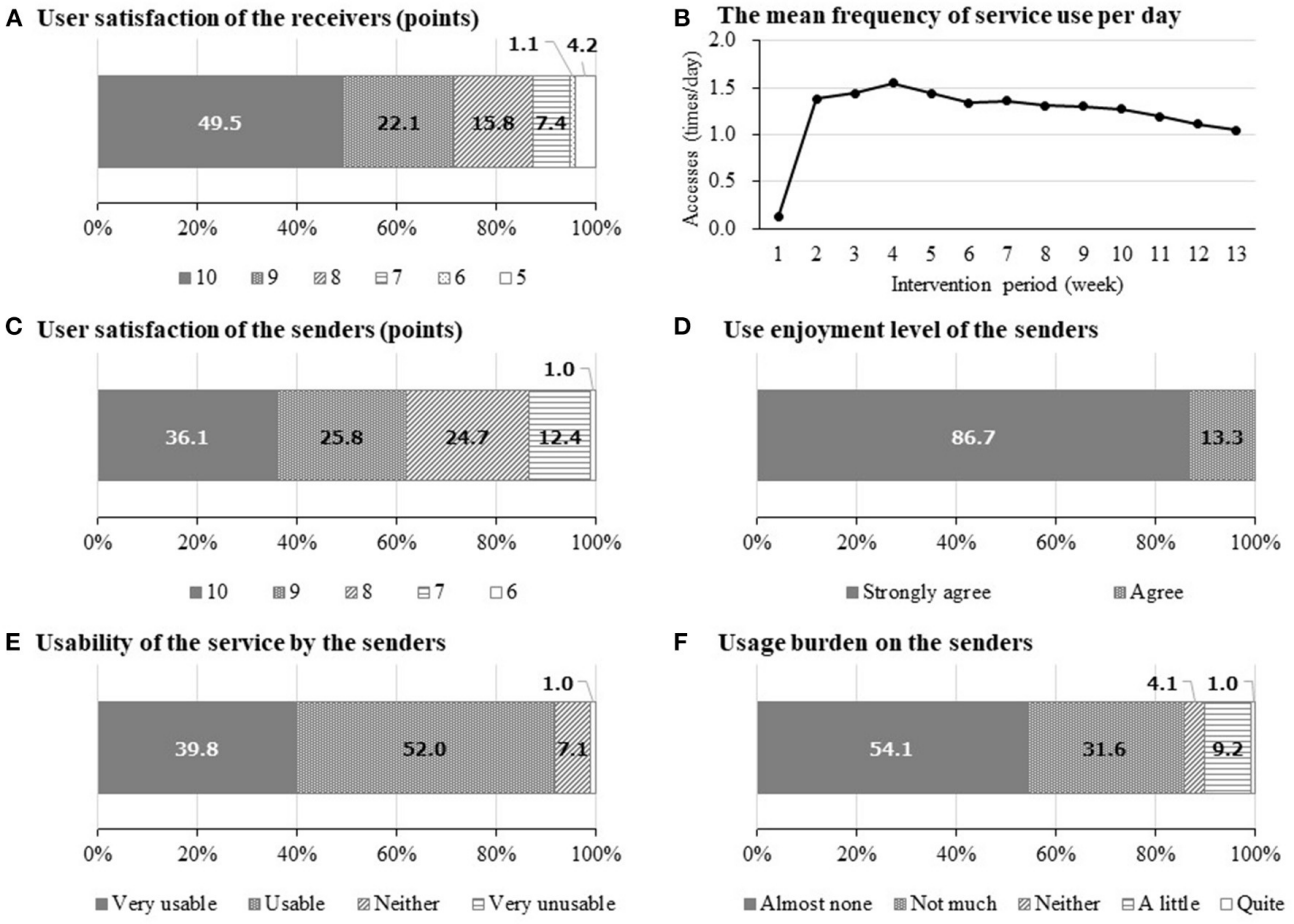
User satisfaction and status of the service during the intervention period. **(A,B)** are data from participants (middle-aged and older adults), and **(C–F)** are data from senders such as separated family members. **(A)** User satisfaction score (0–10 points) by the receivers: mean score (standard deviation: SD) = 9.0 (1.3). **(B)** The mean frequency of service use per day (times/day) by participants during the intervention periods (*n* = 106). **(C)** User satisfaction score (0–10 points) by the senders (*n* = 97): mean score (SD) = 8.84 (1.09). **(D)** Use enjoyment level of the senders (*n* = 98). **(E)** Usability of the service by the senders (*n* = 98). **(F)** Usage burden on the senders (*n* = 98).

Finally, 106 participants completed the three-month intervention. Figure 2A shows the satisfaction of the intervention program. The mean satisfaction score (SD) was 9.0 (1.3) points, with 87.4% of participants scoring ≥ 8 points. Figure 2B shows the mean number of accesses of the “Mago-Channel”; except for the first week of intervention, the number of accesses continued to show ≥ once per day.

Figure 2C shows the satisfaction of the senders, that is, the families not living together; the mean score (SD) was 8.8 (1.1) points, with 86.6% of them scoring ≥ 8 points. Regarding use enjoyment, 100% answered “strongly agree” or “agree” (Figure 2D); regarding service usability, 91.8% answered “very usable” or “usable” (Figure 2E); regarding usage burden, 85.7% answered “almost none” or “not much” (Figure 2F).

For assessment at the end of the intervention, participants “very often” or “often” talking with families living together about the photos/videos received were at 73.0%, those with families not living together were at 67.4%, and those with friends were at 27.1%.

Table 2 shows changes in psychosocial health during the intervention by a linear mixed-effects model. Depressive symptoms remained almost unchanged (β = −0.10, SE = 0.68, *p* = 0.882). Loneliness decreased slightly but did not change significantly (β = −1.02, SE = 0.73, *p* = 0.165). Meanwhile, satisfaction with the relationship of families living together increased significantly (β = 0.71, SE = 0.13, *p* < 0.001). Talking time with families living together increased significantly (β = 9.17, SE = 2.84, *p* = 0.001) and frequency of talking with families not living together also increased (β = 1.15, SE = 0.54, *p* = 0.032). Frequency of talking with friends increased, but not significantly (β = 0.68, SE = 0.54, *p* = 0.212).

**Table 2:** Changes in the psychosocial health indicators during the intervention period.

	**Mean (SD)**				**Effect of time** ^*^		
	**Baseline**	**First** ** month**	**Second** ** month**	**Third** ** month**	**β**	**SE**	***P*-value**
CES-D score	*n =* 92	*n =* 94	*n =* 91	*n =* 83			
	13.1 (7.5)	13.6 (7.1)	13.5 (8.1)	12.5 (7.6)	−0.10	0.68	0.882
UCLA Loneliness Scale score	*n =* 87	*n =* 93	*n =* 92	*n =* 92			
	38.1 (10.0)	36.9 (8.7)	37.2 (9.5)	37.1 (10.2)	−1.02	0.73	0.165
Satisfaction score for the relationship with families living together	*n =* 78	*n =* 82	*n =* 77	*n =* 75			
	7.1 (2.0)	7.4 (1.8)	7.6 (1.7)	7.9 (1.5)	0.71	0.13	<0.001
Talking time with families living together, min/day	*n =* 81	*n =* 84	*n =* 79	*n =* 76			
	70.4 (44.0)	76.1 (39.1)	82.4 (36.8)	83.5 (38.5)	9.17	2.84	0.001
Frequency of talking with families not living together, times/month	*n =* 105	*n =* 96	*n =* 98	*n =* 97			
	8.9 (7.4)	9.1 (7.1)	9.6 (7.3)	10.0 (7.2)	1.15	0.54	0.032
Frequency of talking with friends, times/month	*n =* 106	*n =* 98	*n =* 99	*n =* 97			
	7.2 (7.2)	7.8 (7.6)	8.0 (7.6)	7.8 (7.5)	0.68	0.54	0.212

β, unstandardized coefficient; CES-D, The Center for Epidemiologic Studies Depression Scale; SD, standard deviation; SE, standard error.

* Estimated by a linear mixed-effects model with one unit of intervention period (three months).

Most results of the *post hoc* analyses on psychosocial health showed similar trends to the main analyses; meanwhile, an analysis restricted to those who reported often talking with families not living together about the photos and videos received showed a decrease in loneliness (Supplementary Table 1).

## Discussion

A three-month intervention through ICT-based remote sharing of photos and videos from families not living together showed a low dropout rate and high user satisfaction, indicating adequate feasibility. Although psychological health in middle-aged and older people did not change markedly, their social relationships with families living together and not living together improved.

A notable feature of the services in this intervention is their simplicity, so that anyone, including older people, can easily use them by eliminating unnecessary functions and designs. The device connected to the TV has a built-in telecommunication system, allowing users to connect *via* the Internet without any special settings; they can use it by connecting only two cables (18, 19). Furthermore, the feature to view photos and videos on their TV screen, which is familiar to middle-aged and older people in Japan, rather than on a computer or smartphone, may have led to easier acceptance; it would enable the generation unfamiliar with digital products to having an easy-to-use experience. Additionally, interactions with children and grandchildren have traditionally been normative for middle-aged and older people in Japan, which may have strongly motivated their service uses.

This study intervention improved satisfaction with the relationship of families living together and increased the frequency of talking with families not living together. This intervention may have facilitated conversations with families living together about photos and videos sent, enhancing their relationships. As with similar approaches reported in the past (20–22), photos and videos may be a source of communication with families and friends. Watching the photos and videos sent from families not living together can encourage interactions, including *via* phone and in person. In Japan, the trend toward nuclear families in recent years has led to a rapid increase in the number of people in later adulthood who live apart from their children and grandchildren (27), and their relationships with them are becoming weaker. An informal social connection involving sharing photos and videos with people outside the family living together, including their children and grandchildren, may encourage social interaction. Future research, such as exploration of user experiences using mixed-methods studies, is needed to further develop intervention design.

However, this intervention did not reduce depressive symptoms and loneliness and, thus, might not have enough intensity to change the psychological status. Meanwhile, the *post hoc* analysis indicated that those who often talked with their families not living together about the photos and videos felt reduced loneliness. Although the multiple testing issue should be noticed, this service use might improve psychological health if it can promote interactions sufficiently with families not living together. Further research is required to examine this hypothesis, including what level of interaction is appropriate.

This study recommended that senders (family members not living together) post photos and videos at least once a week; 9.4% of participants received them <10 of the 12 weeks of intervention periods (Supplementary Figure 1), which perhaps suggests that some participants had insufficient intervention. However, the *post hoc* analysis limited to those who continuously received photos and videos showed almost the same results (Supplementary Table 1). We need further studies to explore the factors that increase the effects of interventions.

This study has several limitations. First, this study did not have a control group, and therefore, we did not test its effects. Further studies, including randomized control trials, were required. Second, the intervention period coincided with the COVID-19 outbreak duration (7). Social behavior restrictions to prevent infections may externally alter participants' psychosocial health. Additionally, we cannot exclude the possibility that the intervention behaved more acceptably because of restraint in epidemic periods. Third, many of the assessment scales (e.g., satisfaction) used in this study have not been validated. Because there was a possibility of measurement error, it is required to evaluate using validated scales. Additionally, we converted the responses regarding the time and frequency of talking into continuous variables based on previous studies (25, 26); however, the validity of this operation should be further warranted. The self-report question in this non-blind intervention may have caused measurement errors, which may result in information bias. Therefore, further research is necessary using more valid measurement methods, such as observation. Fourth, although this study was able to evaluate the feasibility in the relatively short period of 3 months, in the long term this is unknown. Therefore, further investigations based on longer intervention periods are needed. Fifth, participants may not necessarily have the same characteristics as typical middle-aged and older people. Considering that this intervention required sending of photos and videos from others, that is, the families not living together, study participants were limited to those well-related others. Additionally, this study's results came from middle-aged and older people in Japan, which is different from Western culture. Therefore, the generalizability and transportability of our results should be noted.

In conclusion, this study showed adequate feasibility of intervention on the psychosocial health of middle-aged and older adults through remote sharing of photos and videos from families not living together. To establish an age-friendly ICT-based new approach to their psychosocial health, investigating the efficacy through further studies is required.

## Data availability statement

All our datasets have ethical or legal restrictions for public deposition because of the inclusion of sensitive information about the participants. Requests to access the datasets should be directed to TN, noguchi@ncgg.go.jp.

## Ethics statement

The studies involving human participants were reviewed and approved by Research Ethics Committee of the National Center for Geriatrics and Gerontology. The patients/participants provided their written informed consent to participate in this study.

## Author contributions

TN conceptualized and designed the study, participated in data collection, analyzed the data, and drafted and revised the manuscript. MS conceptualized the study, supported the interpretation of the results, and reviewed and critically revised the manuscript. TS conceptualized and designed the study, supported data collection, interpreted the results, and reviewed and critically revised the manuscript. All authors approved the submission of the final manuscript.

## Funding

This study was supported by Joint Research Funding of the National Center for Geriatrics and Gerontology and Chikaku Inc. This study was also supported by the Japan Society for the Promotion of Science (JSPS) KAKENHI Grant Numbers 21K17322. The funder was not involved in the study design, collection, analysis, interpretation of data, and the writing of this article or the decision to submit it for publication.

## Conflict of interest

MS was employed by Chikaku Inc. The remaining authors declare that the research was conducted in the absence of any commercial or financial relationships that could be construed as a potential conflict of interest.

## Publisher's note

All claims expressed in this article are solely those of the authors and do not necessarily represent those of their affiliated organizations, or those of the publisher, the editors and the reviewers. Any product that may be evaluated in this article, or claim that may be made by its manufacturer, is not guaranteed or endorsed by the publisher.
