# Supplementary material for: An approach to psychosocial health among middle-aged and older people by remote sharing of photos and videos from family members not living together: A feasibility study
Source: Front Public Health. 2022 Nov 10;10:962977. doi: 10.3389/fpubh.2022.962977 (PMC9686400; doi:10.3389/fpubh.2022.962977)
Supplement: Supplementary file 1 [file Table_1.DOCX]

Supplementary Material

# Supplementary Table

**Supplementary Table 1. Changes in the psychosocial health indicators during the intervention period, post hoc analyses**

|  | Mean (SD) | | | |  | Effect of time* | | |
| --- | --- | --- | --- | --- | --- | --- | --- | --- |
|  | Baseline | First month | Second month | Third month |  | β | SE | P-value |
| **Those without mental illness and taking psychotropic drugs (n = 97)** | | | |  |  |  |  |  |
| CES-D score | n = 84 | n = 87 | n = 85 | n = 76 |  |  |  |  |
|  | 12.3 (6.7) | 13.3 (6.8) | 13.3 (8.2) | 12.1 (7.4) |  | 0.14 | 0.71 | 0.847 |
| UCLA Loneliness Scale score | n = 80 | n = 86 | n = 87 | n = 84 |  |  |  |  |
|  | 37.7 (9.7) | 36.8 (8.5) | 37.2 (9.4) | 36.9 (1.0) |  | -0.86 | 0.74 | 0.249 |
| Satisfaction score for the relationship with families living together | n = 70 | n = 74 | n = 70 | n = 68 |  |  |  |  |
|  | 7.2 (1.9) | 7.5 (1.7) | 7.6 (1.6) | 7.9 (1.4) |  | 0.71 | 0.13 | < 0.001 |
| Talking time with families living together, min/day | n = 73 | n = 76 | n = 72 | n = 70 |  |  |  |  |
|  | 72.7 (42.8) | 78.2 (37.8) | 83.1 (36.7) | 86.4 (36.9) |  | 9.30 | 3.01 | 0.002 |
| Frequency of talking with families not living together, times/month | n = 96 | n = 88 | n = 90 | n = 89 |  |  |  |  |
|  | 9.1 (7.4) | 9.4 (7.1) | 9.9 (7.3) | 10.2 (7.1) |  | 1.08 | 0.57 | 0.059 |
| Frequency of talking with friends, times/month | n = 97 | n = 90 | n = 91 | n = 89 |  |  |  |  |
|  | 7.4 (7.2) | 7.6 (7.4) | 8.0 (7.7) | 7.9 (7.3) |  | 0.62 | 0.56 | 0.267 |
|  |  |  |  |  |  |  |  |  |
| **Those without low vision (n = 90)** |  |  |  |  |  |  |  |  |
| CES-D score | n = 80 | n = 81 | n = 76 | n = 72 |  |  |  |  |
|  | 11.9 (6.7) | 13.3 (7.0) | 12.1 (6.7) | 12.3 (7.7) |  | 0.23 | 0.71 | 0.748 |
| UCLA Loneliness Scale score | n = 75 | n = 80 | n = 79 | n = 78 |  |  |  |  |
|  | 37.8 (9.9) | 36.6 (8.6) | 36.8 (9.4) | 37.0 (10.5) |  | -0.88 | 0.79 | 0.267 |
| Satisfaction score for the relationship with families living together | n = 65 | n = 70 | n = 66 | n = 64 |  |  |  |  |
|  | 7.4 (1.9) | 7.5 (1.7) | 7.7 (1.6) | 8.0 (1.5) |  | 0.56 | 0.14 | < 0.001 |
| Talking time with families living together, min/day | n = 67 | n = 71 | n = 67 | n = 64 |  |  |  |  |
|  | 74.6 (42.3) | 76.1 (39.7) | 82.8 (37.4) | 84.6 (38.8) |  | 7.25 | 2.84 | 0.011 |
| Frequency of talking with families not living together, times/month | n = 90 | n = 82 | n = 83 | n = 83 |  |  |  |  |
|  | 8.5 (7.3) | 9.0 (7.1) | 8.9 (6.9) | 9.6 (7.1) |  | 0.97 | 0.55 | 0.078 |
| Frequency of talking with friends, times/month | n = 90 | n = 84 | n = 84 | n = 83 |  |  |  |  |
|  | 7.0 (7.0) | 7.8 (7.5) | 7.7 (7.4) | 7.7 (7.2) |  | 0.74 | 0.58 | 0.202 |
|  |  |  |  |  |  |  |  |  |
| **Those living alone (n = 22)** |  |  |  |  |  |  |  |  |
| CES-D score | n = 18 | n = 17 | n = 18 | n = 16 |  |  |  |  |
|  | 12.8 (7.7) | 12.1 (5.4) | 10.9 (4.6) | 11.4 (6.8) |  | -1.11 | 1.25 | 0.379 |
| UCLA Loneliness Scale score | n = 15 | n = 15 | n = 18 | n = 17 |  |  |  |  |
|  | 39.9 (10.6) | 39.2 (10.6) | 38.1 (11.3) | 39.5 (11.8) |  | -1.84 | 1.58 | 0.251 |
| Satisfaction score for the relationship with families living together | – | – | – | – |  | – | – | – |
|  |  |  |  |  |  |  |  |  |
| Talking time with families living together, min/day | – | – | – | – |  | – | – | – |
|  |  |  |  |  |  |  |  |  |
| Frequency of talking with families not living together, times/month | n = 22 | n = 16 | n = 19 | n = 19 |  |  |  |  |
|  | 12.3 (7.2) | 13.0 (6.7) | 12.3 (7.1) | 12.2 (7.3) |  | -0.12 | 1.22 | 0.925 |
| Frequency of talking with friends, times/month | n = 22 | n = 17 | n = 19 | n = 19 |  |  |  |  |
|  | 7.2 (7.8) | 9.4 (8.8) | 11.8 (9.1) | 11.3 (8.6) |  | 4.05 | 1.37 | 0.004 |
|  |  |  |  |  |  |  |  |  |
| **Those aged ≥ 75 years (n = 52)** |  |  |  |  |  |  |  |  |
| CES-D score | n = 43 | n = 43 | n = 42 | n = 38 |  |  |  |  |
|  | 14.9 (7.2) | 15.6 (7.0) | 14.6 (9.1) | 13.2 (7.8) |  | -0.96 | 1.01 | 0.384 |
| UCLA Loneliness Scale score | n = 41 | n = 42 | n = 43 | n = 43 |  |  |  |  |
|  | 36.5 (9.1) | 35.6 (7.4) | 35.2 (9.2) | 34.9 (8.6) |  | -1.90 | 1.03 | 0.066 |
| Satisfaction score for the relationship with families living together | n = 36 | n = 38 | n = 35 | n = 32 |  |  |  |  |
|  | 7.4 (1.9) | 7.6 (1.7) | 7.9 (1.7) | 8.2 (1.4) |  | 0.69 | 0.21 | 0.001 |
| Talking time with families living together, min/day | n = 38 | n = 39 | n = 37 | n = 33 |  |  |  |  |
|  | 69.1 (47.4) | 73.5 (41.1) | 81.9 (37.9) | 86.8 (37.5) |  | 10.02 | 4.30 | 0.022 |
| Frequency of talking with families not living together, times/month | n = 51 | n = 46 | n = 48 | n = 46 |  |  |  |  |
|  | 9.6 (6.7) | 10.2 (6.6) | 10.8 (7.1) | 11.3 (6.7) |  | 1.56 | 0.78 | 0.046 |
| Frequency of talking with friends, times/month | n = 52 | n = 47 | n = 48 | n = 46 |  |  |  |  |
|  | 7.8 (7.2) | 8.3 (7.9) | 8.9 (7.7) | 8.9 (7.6) |  | 1.33 | 0.87 | 0.132 |
|  |  |  |  |  |  |  |  |  |
| **Men (n = 47)** |  |  |  |  |  |  |  |  |
| CES-D score | n = 41 | n = 42 | n = 58 | n = 37 |  |  |  |  |
|  | 12.1 (5.5) | 13.4 (7.8) | 12.9 (8.5) | 11.7 (7.8) |  | -0.41 | 0.89 | 0.646 |
| UCLA Loneliness Scale score | n = 38 | n = 43 | n = 39 | n = 42 |  |  |  |  |
|  | 38.8 (9.1) | 35.8 (7.6) | 36.6 (9.0) | 36.7 (10.0) |  | -1.87 | 1.18 | 0.117 |
| Satisfaction score for the relationship with families living together | n = 43 | n = 43 | n = 43 | n = 42 |  |  |  |  |
|  | 7.8 (1.5) | 7.9 (1.5) | 8.1 (1.5) | 8.3 (1.4) |  | 0.44 | 0.17 | 0.011 |
| Talking time with families living together, min/day | n = 44 | n = 44 | n = 43 | n = 43 |  |  |  |  |
|  | 82.2 (43.1) | 82.2 (37.2) | 90.7 (35.4) | 92.4 (36.4) |  | 10.34 | 3.76 | 0.007 |
| Frequency of talking with families not living together, times/month | n = 46 | n = 45 | n = 42 | n = 44 |  |  |  |  |
|  | 7.1 (6.7) | 8.0 (7.0) | 8.2 (7.1) | 9.0 (7.1) |  | 1.56 | 0.84 | 0.068 |
| Frequency of talking with friends, times/month | n = 47 | n = 45 | n = 43 | n = 44 |  |  |  |  |
|  | 6.1 (6.3) | 6.5 (6.8) | 5.7 (6.4) | 5.6 (6.1) |  | -0.47 | 0.81 | 0.564 |
|  |  |  |  |  |  |  |  |  |
| **Those who rarely use e-mail or social networking services at baseline (n = 48)** | | | | |  |  |  |  |
| CES-D score | n = 40 | n = 38 | n = 37 | n = 34 |  |  |  |  |
|  | 15.1 (9.2) | 15.5 (8.1) | 15.4 (9.8) | 13.9 (8.5) |  | -0.15 | 1.26 | 0.905 |
| UCLA Loneliness Scale score | n = 39 | n = 38 | n = 36 | n = 40 |  |  |  |  |
|  | 38.0 (10.7) | 36.5 (8.5) | 37.2 (10.1) | 37.0 (9.8) |  | -0.83 | 1.07 | 0.437 |
| Satisfaction score for the relationship with families living together | n = 40 | n = 37 | n = 37 | n = 36 |  |  |  |  |
|  | 7.1 (2.1) | 7.4 (1.6) | 7.7 (1.7) | 7.9 (1.4) |  | 0.90 | 0.22 | < 0.001 |
| Talking time with families living together, min/day | n = 37 | n = 37 | n = 36 | n = 34 |  |  |  |  |
|  | 67.3 (46.8) | 76.6 (38.0) | 81.9 (34.4) | 83.8 (38.4) |  | 9.63 | 4.56 | 0.035 |
| Frequency of talking with families not living together, times/month | n = 47 | n = 40 | n = 42 | n = 42 |  |  |  |  |
|  | 6.9 (6.9) | 7.6 (6.5) | 9.1 (7.5) | 9.0 (7.3) |  | 2.49 | 0.79 | 0.002 |
| Frequency of talking with friends, times/month | n = 48 | n = 42 | n = 43 | n = 42 |  |  |  |  |
|  | 6.0 (6.7) | 7.2 (7.5) | 7.4 (7.4) | 7.9 (7.4) |  | 1.75 | 0.94 | 0.065 |
|  |  |  |  |  |  |  |  |  |
| **Those who interact less with families not living together at baseline (n = 27)** | | | |  |  |  |  |  |
| CES-D score | n = 25 | n = 26 | n = 22 | n = 20 |  |  |  |  |
|  | 13.1 (8.5) | 13.7 (6.2) | 12.5 (5.8) | 11.6 (5.2) |  | -1.77 | 1.16 | 0.131 |
| UCLA Loneliness Scale score | n = 23 | n = 26 | n = 25 | n = 24 |  |  |  |  |
|  | 41.0 (10.8) | 40.8 (8.6) | 39.2 (9.7) | 40.5 (10.5) |  | -1.13 | 1.25 | 0.370 |
| Satisfaction score for the relationship with families living together | n = 24 | n = 24 | n = 25 | n = 23 |  |  |  |  |
|  | 6.8 (1.8) | 7.2 (1.7) | 7.3 (1.4) | 7.8 (1.4) |  | 0.85 | 0.25 | < 0.001 |
| Talking time with families living together, min/day | n = 24 | n = 25 | n = 25 | n = 23 |  |  |  |  |
|  | 60.6 (43.9) | 70.2 (38.2) | 69.0 (38.2) | 77.6 (40.6) |  | 14.21 | 4.94 | 0.005 |
| Frequency of talking with families not living together, times/month | n = 27 | n = 27 | n = 26 | n = 25 |  |  |  |  |
|  | 1.2 (0.6) | 3.0 (3.1) | 2.6 (2.8) | 2.8 (2.8) |  | 1.39 | 0.60 | 0.023 |
| Frequency of talking with friends, times/month | n = 27 | n = 27 | n = 27 | n = 25 |  |  |  |  |
|  | 4.5 (5.8) | 5.4 (6.8) | 6.2 (7.4) | 3.6 (5.9) |  | 0.40 | 0.90 | 0.656 |
|  |  |  |  |  |  |  |  |  |
| **Those who often talked with families living together about the photos/videos received (n = 65)** | | | | | | |  |  |
| CES-D score | n = 57 | n = 60 | n = 60 | n = 56 |  |  |  |  |
|  | 12.7 (7.0) | 14.3 (7.8) | 14.4 (8.7) | 12.6 (8.2) |  | 0.22 | 0.89 | 0.802 |
| UCLA Loneliness Scale score | n = 54 | n = 61 | n = 59 | n = 63 |  |  |  |  |
|  | 37.4 (9.8) | 36.0 (7.8) | 36.8 (8.8) | 35.6 (9.3) |  | -1.27 | 0.94 | 0.178 |
| Satisfaction score for the relationship with families living together | n = 59 | n = 63 | n = 63 | n = 63 |  |  |  |  |
|  | 7.2 (1.9) | 7.6 (1.7) | 7.7 (1.7) | 8.0 (1.4) |  | 0.75 | 0.15 | < 0.001 |
| Talking time with families living together, min/day | n = 60 | n = 63 | n = 63 | n = 64 |  |  |  |  |
|  | 79.3 (40.7) | 86.0 (32.8) | 89.5 (32.8) | 90.5 (34.0) |  | 10.38 | 3.30 | 0.002 |
| Frequency of talking with families not living together, times/month | n = 64 | n = 64 | n = 63 | n = 65 |  |  |  |  |
|  | 8.8 (7.4) | 8.7 (7.2) | 9.6 (7.4) | 10.2 (7.2) |  | 1.44 | 0.68 | 0.036 |
| Frequency of talking with friends, times/month | n = 65 | n = 64 | n = 64 | n = 65 |  |  |  |  |
|  | 7.6 (6.9) | 7.3 (7.5) | 7.2 (6.9) | 7.6 (7.2) |  | -0.10 | 0.69 | 0.883 |
|  |  |  |  |  |  |  |  |  |
| **Those who often talked with families not living together about the photos/videos received (n = 64)** | | | | | | |  |  |
| CES-D score | n = 56 | n = 57 | n = 59 | n = 52 |  |  |  |  |
|  | 12.6 (6.9) | 14.4 (8.0) | 13.9 (8.7) | 12.5 (8.4) |  | 0.21 | 0.90 | 0.819 |
| UCLA Loneliness Scale score | n = 51 | n =57 | n = 57 | n = 61 |  |  |  |  |
|  | 38.2 (10.3) | 36.3 (8.6) | 37.0 (9.9) | 36.0 (9.7) |  | -2.23 | 0.93 | 0.018 |
| Satisfaction score for the relationship with families living together | n = 46 | n = 52 | n = 47 | n = 47 |  |  |  |  |
|  | 7.2 (2.1) | 7.4 (1.8) | 7.8 (1.7) | 8.0 (1.5) |  | 0.77 | 0.19 | < 0.001 |
| Talking time with families living together, min/day | n = 47 | n = 52 | n = 48 | n = 48 |  |  |  |  |
|  | 71.8 (44.5) | 79.6 (38.0) | 90.0 (33.9) | 87.5 (36.1) |  | 12.39 | 3.94 | 0.002 |
| Frequency of talking with families not living together, times/month | n = 63 | n = 60 | n = 63 | n = 64 |  |  |  |  |
|  | 10.1 (7.8) | 10.3 (7.1) | 10.6 (7.3) | 11.6 (7.2) |  | 1.36 | 0.70 | 0.054 |
| Frequency of talking with friends, times/month | n = 64 | n = 61 | n = 63 | n = 64 |  |  |  |  |
|  | 7.6 (7.5) | 8.6 (8.0) | 7.6 (7.4) | 8.1 (7.6) |  | 0.17 | 0.67 | 0.797 |
|  |  |  |  |  |  |  |  |  |
| **Those who often talked with friends about the photos/videos received (n = 26)** | | | | |  |  |  |  |
| CES-D score | n = 19 | n = 21 | n = 22 | n = 21 |  |  |  |  |
|  | 13.7 (8.8) | 16.1 (10.1) | 16.3 (10.8) | 16.0 (9.8) |  | 3.01 | 1.89 | 0.177 |
| UCLA Loneliness Scale score | n = 19 | n = 22 | n = 20 | n = 24 |  |  |  |  |
|  | 33.8 (7.9) | 33.8 (7.7) | 32.8 (10.0) | 33.6 (8.8) |  | -1.72 | 1.48 | 0.248 |
| Satisfaction score for the relationship with families living together | n = 18 | n = 20 | n = 17 | n = 18 |  |  |  |  |
|  | 6.7 (2.4) | 7.3 (2.2) | 7.9 (1.9) | 8.1 (1.7) |  | 1.35 | 0.36 | < 0.001 |
| Talking time with families living together, min/day | n = 19 | n = 20 | n = 18 | n = 18 |  |  |  |  |
|  | 58.4 (44.7) | 72.0 (36.3) | 85.8 (40.5) | 81.7 (40.6) |  | 21.12 | 6.86 | 0.003 |
| Frequency of talking with families not living together, times/month | n = 25 | n = 24 | n = 25 | n = 26 |  |  |  |  |
|  | 11.3 (6.0) | 12.3 (6.1) | 13.2 (6.9) | 12.6 (6.1) |  | 1.32 | 1.13 | 0.247 |
| Frequency of talking with friends, times/month | n = 26 | n = 25 | n = 25 | n = 25 |  |  |  |  |
|  | 10.6 (7.7) | 12.2 (8.9) | 12.4 (7.8) | 12.6 (7.4) |  | 1.86 | 1.36 | 0.176 |
|  |  |  |  |  |  |  |  |  |
| **Those who received photos/videos continuously during the intervention period (n = 54)** | | | | |  |  |  |  |
| CES-D score | n = 46 | n = 44 | n = 47 | n = 43 |  |  |  |  |
|  | 12.4 (7.6) | 13.6 (7.8) | 13.7 (9.8) | 11.3 (7.8) |  | -0.55 | 0.95 | 0.561 |
| UCLA Loneliness Scale score | n = 44 | n = 46 | n = 47 | n = 50 |  |  |  |  |
|  | 38.4 (10.4) | 36.2 (8.8) | 36.0 (9.3) | 36.3 (11.1) |  | -1.79 | 1.02 | 0.082 |
| Satisfaction score for the relationship with families living together | n = 41 | n = 43 | n = 41 | n = 40 |  |  |  |  |
|  | 7.2 (2.1) | 7.2 (1.9) | 7.8 (1.7) | 7.8 (1.6) |  | 0.67 | 0.15 | < 0.001 |
| Talking time with families living together, min/day | n = 42 | n = 43 | n = 41 | n = 42 |  |  |  |  |
|  | 79.3 (44.6) | 81.3 (40.5) | 89.3 (35.7) | 88.2 (39.5) |  | 7.52 | 3.72 | 0.045 |
| Frequency of talking with families not living together, times/month | n = 53 | n = 50 | n = 51 | n = 52 |  |  |  |  |
|  | 9.1 (7.6) | 9.1 (7.6) | 10.2 (7.3) | 10.7 (7.3) |  | 1.77 | 0.75 | 0.020 |
| Frequency of talking with friends, times/month | n = 54 | n = 51 | n = 52 | n = 52 |  |  |  |  |
|  | 7.5 (7.8) | 8.6 (8.2) | 8.6 (8.0) | 8.4 (7.9) |  | 0.64 | 0.77 | 0.411 |

β, unstandardized coefficient; CES-D, The Center for Epidemiologic Studies Depression Scale; SD, standard deviation; SE, standard error.

*Estimated by a linear mixed-effects model with one unit of intervention period (three months).

# Supplementary Figure


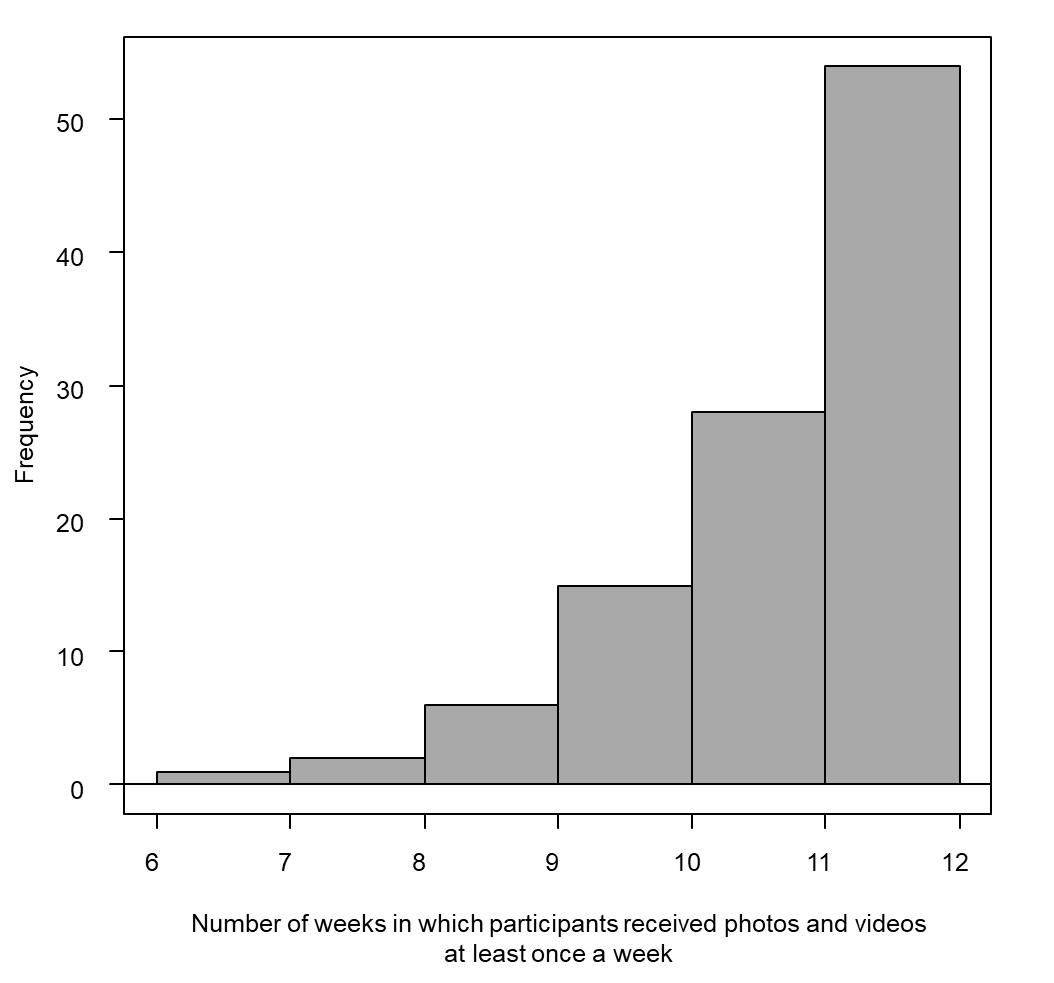


**Supplementary Figure 1. Distribution of the number of weeks in which participants received photos and videos at least once a week during the 12-week intervention period.** Mean (standard deviation) = 11.2 (1.1) weeks (min = 6, max = 12).
